# Metabolomic Markers in Tongue-Coating Samples from Damp Phlegm Pattern Patients of Coronary Heart Disease and Chronic Renal Failure

**DOI:** 10.1155/2019/4106293

**Published:** 2019-09-08

**Authors:** Yiming Hao, Xue Yuan, Jin Yan, Minh Pham, Dekai Rohlsen, Peng Qian, Feng Cheng, Yiqin Wang

**Affiliations:** ^1^Shanghai Key Laboratory of Health Identification and Assessment/Laboratory of TCM Four Diagnostic Information, Shanghai University of Traditional Chinese Medicine, Shanghai 201203, China; ^2^Shanghai Haohai Biological Technology Co., Ltd., Shanghai 200052, China; ^3^Jiangsu Province Hospital of Chinese Medicine, Nanjing, Jiangsu Province 210009, China; ^4^Department of Mathematics and Statistics, University of South Florida, Tampa, FL 33620, USA; ^5^Department of Pharmaceutical Sciences, College of Pharmacy, University of South Florida, Tampa, FL 33612, USA

## Abstract

In this paper, we used tongue coating to obtain metabolites in patients with coronary heart disease (CHD) and chronic renal failure (CRF). The metabolites were analyzed to discover the substance that serves as the underlying basis of the damp phlegm pattern. This analysis is based on the Traditional Chinese Medicine (TCM) theory of “different diseases have the same pattern.” The metabolic spectrum was obtained through the Gas Chromatography Mass Spectrometry coupling techniques and analyzed by searching the METLIN and HMDB databases. Some metabolites related to amino acids and glucose metabolism were identified in the tongue-coating samples from damp phlegm pattern patients by comparing them to nondamp phlegm pattern patients and healthy subjects. In addition, there were five common metabolites in the tongue-coating samples from CHD damp phlegm pattern patients compared to CRF damp phlegm pattern patients, which allowed us to understand the theory of “different diseases have the same pattern.” In the future, the metabolites identified in this study may be used as noninvasive and convenient biomarkers to distinguish the damp phlegm pattern of CHD and CRF patients.

## 1. Introduction

Coronary heart disease (CHD) is a disease in which an atherosclerotic lesion of the coronary artery causes stenosis or obstruction of the vascular cavity, which could lead to myocardial ischemia, anoxia, or necrosis. The incidence rate of CHD has increased significantly due to the rapid change of the economy, the alteration of lifestyle, and the tendency of population aging. The morbidity and mortality rates of CHD also increased year by year, and the trend is significant among younger generations. Chronic renal failure (CRF) is a clinical syndrome of chronic progressive renal parenchyma damage caused by various reasons. The main clinical manifestations were atrophy of the kidney; inability to maintain basic functions; retention of metabolites; imbalance of water, electrolyte, and acid base; and systemic involvement. The incidence rate of CRF has increased at a noticeable rate. Nowadays, millions of people worldwide die of complications related to CRF each year [1]. The prevention and control of CRF has become a challenge for researchers and scientists around the world.

The damp phlegm pattern is the definition of a certain kind of disease according to the symptoms and signs of patients based on Traditional Chinese Medicine (TCM) theory. According to TCM theory, the damp phlegm pattern is due to internal organ dysfunction, which hinders the movement of water in the body; thus, the water accumulates to form dampness and phlegm. The patients with damp phlegm pattern mainly have full and heavy sensation in the body, greasy tongue coating, and slippery pulse. At present, the damp phlegm pattern as one of the most common TCM patterns has been included in the International Classification of Diseases, 11th Revision (ICD-11), compiled by the World Health Organization (WHO) [2]. One of the TCM theories named “different diseases have the same pattern” means that patients with different diseases may have the same clinical manifestations caused by a common pathogenesis. And the damp phlegm pattern has been considered a common pattern in CHD and CRF.

After analyzing the CHD-related pattern literature in TCM collected over the past 40 years, we found that the percentage of patients with the damp phlegm pattern among 34,640 patients with CHD was 13.48% [3]. Some studies found that the damp phlegm pattern was also common in the progression of CRF [4]. However, the molecular mechanism of the damp phlegm pattern in patients with CHD and CRF is still unknown.

As an important part of inspection and one of the TCM-unique diagnostics, tongue diagnosis is an approach in TCM clinical practice by observing the changes of tongue property and tongue coating to understand the physiological functions and pathological changes of the body. Therefore, the tongue coating of patients with the damp phlegm pattern may have something different from those of healthy people, which may be selected as the disease markers of the damp phlegm pattern. In previous studies, our group has identified proteins in the tongue coating and serum of patients with CHD and CRF showing the damp phlegm pattern by proteomic technique [5–8]. These studies provided new insights into the damp phlegm pattern of TCM at the protein level.

In this study, we investigated the metabolites in the tongue coating of damp phlegm pattern patients of CHD and CRF by using metabolomic methods that can obtain large amounts of data about the changes of metabolites in a systemic level [9, 10]. The collection of tongue-coating samples is noninvasive and convenient. Currently, the main metabolomic approaches include Nuclear Magnetic Resonance (NMR), Gas Chromatography Mass Spectrometry (GC-MS), and Liquid Chromatography Mass Spectrometry (LC-MS). In the three methods, the GC-MS test has higher sensitivity and lower cost. Therefore, we applied GC-MS in this study to investigate the metabolomic markers of the damp phlegm pattern of CHD and CRF and the nature of the TCM theory “different diseases have the same pattern” through analyzing the tongue-coating metabolites of patients with the damp phlegm pattern in CHD and CRF.

## 2. Materials and Methods

### 2.1. Patients

This is a case-control study. Fifty-eight patients with CHD who were diagnosed with mild to moderate coronary artery stenosis were selected from the Municipal Hospital of Traditional Chinese Medicine affiliated with Shanghai University of Traditional Chinese Medicine. Sixty-two patients with CRF caused by acute or chronic glomerular nephritis who were diagnosed at the chronic kidney disease (CKD) 1-3 phases were selected from the Longhua Hospital and Shuguang Hospital affiliated with Shanghai University of TCM. All patients were admitted to the two hospitals from September 2011 to July 2012. Tongue coating of patients were collected (see Experimental Methods) and the clinical questionnaire forms for CHD and CRF were filled. Through the guidance of two TCM doctors, patients with the two types of diseases were divided into two groups in accordance with the standard of TCM diagnosis (see Section 2.3 in detail), the damp phlegm pattern and the nondamp phlegm pattern group. Twenty-five healthy staff members in the Shanghai University of TCM without any organic lesion in the system were selected as controls, and their tongue-coating samples were collected. Based on the study of Nyamundanda et al., our sample size can achieve the false discovery rate (FDR) at 5% for a metabolomic study [11].

There was no significant difference (*P* > 0.05) between the groups at the aspects of gender and age, so the data from these groups were comparable (Table 1).

### 2.2. Ethics Approval

The study was approved by the Ethics Committee of Shanghai University of Traditional Chinese Medicine in China in September 2011 and performed in accordance with the Declaration of Helsinki. All the subjects signed informed consent forms verifying consent and compliance.

### 2.3. The Standard of Diagnosis, Admission, Elimination, and Rejection

The diagnostic criteria of CHD are referenced from “Nomenclature and Criteria for Diagnosis of Ischemic Heart Disease” [12]. The diagnostic criteria of CRF are referenced from the “Kidney Disease Outcome Quality Initiative” [13].

The diagnostic criteria of the TCM pattern in CHD and CRF are set according to the “Guiding Principles of Clinical Research on New Drugs of TCM” [14]. The diagnostic criteria of the TCM pattern in CHD and CRF are as follows.

Damp phlegm pattern in CHD

1. patient feels chest is suffocating
2. patient feels pain in chest, and pain may appear on the patient's shoulder and back
3. patient has shortness of breath and/or rush of wheezing
4. patient has high fat and/or much sputum
5. patient has heavy sensation in the limbs and body
6. patient's tongue coating is greasy or slippery, and pulse is slippery

A patient with the damp phlegm pattern in CHD should have one of the first and second symptoms; any two of the third, fourth, and fifth symptoms; and the last symptoms.

Nondamp phlegm pattern in CHD

1. patient has none of the above symptoms of the damp phlegm pattern in CHD

Damp phlegm pattern in CRF

1. patient is nauseous and vomiting, has anorexia, and has heavy sensation in the limbs and body
2. patient feels epigastric and/or abdomen is full
3. patient feels mouth is sticky
4. patient's tongue coating is greasy
5. patient has none of the above symptoms but only has edema and/or pleural effusion and/or ascites

A patient with the damp phlegm pattern in CRF should have the first symptom and one of the second, third, and fourth symptoms, or only the fifth symptom.

Nondamp phlegm pattern in CRF

1. patient has none of the above symptoms of the damp phlegm pattern in CRF.

The admission criteria for patients with CHD/CRF are based on the criteria of Western medicine and TCM pattern diagnosis for CHD/CRF. The patients' age ranges from 25 to 75.

The elimination criteria of CHD are described as follows:

1. Patients associated with heart failure
2. Patients with lung, kidney, blood, endocrine, metabolism, and gastrointestine primary diseases or mental illness
3. Patients who are pregnant or breastfeeding
4. Patients with an allergic constitution

The elimination criteria of CRF include the following:

1. Patients with heart, liver, lung, endocrine, blood, and metabolism primary diseases and severe gastrointestine primary diseases or mental illness
2. Patients with CRF caused by the extrarenal diseases
3. Patients with CRF and dialysis therapy is required
4. Patients who are pregnant or breastfeeding
5. Patients with allergic constitution or with drug allergy

The criteria of rejection include the following:

1. Patients without complete clinical data, either due to incomplete collection or due to data missing

### 2.4. Experimental Methods

#### 2.4.1. The Collection of Tongue-Coating Samples

The method for the collection of tongue-coating samples was developed by our group [15]. Before collection, patients rinsed the mouth once or twice with 200 mL of 0.9% saline. We scraped the appropriate amount of tongue-coating samples with a stainless-steel spoon sterilized by autoclaving from the surface areas of the tongue where the coating is thickest. The samples were put into the sterilized EP tubes with 2 mL saline, sealed, and stored in the -80°C freezer for future usage.

#### 2.4.2. The Counting of Tongue-Coating Samples

In order to accurately quantify the tongue-coating samples after the reaction, we performed the cell counting. Samples were thawed by being placed into water at 0°C. The solution of tongue coatings was centrifuged at 2000 rpm/min for 10 minutes. The supernatant was collected for filling and preservation. The pellet was resuspended and washed with 1.5 mL saline followed by centrifuged again at 2000 rpm/min for 10 minutes. The supernatant was discarded, and the pellet was washed again with 1.5 mL saline. After centrifugation, the pellet was resuspended with 2 mL saline with pipetting. A certain amount of the tongue-coating solution was diluted in proportion and counted with the hemocytometer under the microscope at low magnification.

#### 2.4.3. Sample Derivative Processing

For sample derivation, proteins were first removed by lyophilization and then processed with the silane derivative method [16].

#### 2.4.4. Metabolite Detection by GC-MS

Tongue-coating metabolite detection was performed using the gas chromatography-quadrupole mass spectrometer (Thermo Trace DSQ, USA, Thermo Fisher Scientific).

The chromatographic condition

1. the specifications of the chromatographic column were 60 m × 0.25 mmi.d.×0.25 *μ*m, TR-5MS
2. the initial temperature was set to 80°C and kept for 2 minutes before being ramped up to 140°C at a rate of 10°C/min. The temperature then continued to be ramped up to 240°C at a rate of 4°C/min and to 280°C at a rate of 10°C/min, then held at 280°C for 10 minutes
3. the carrier gas was helium with a flow rate of 1.0 mL/min
4. the temperature of the injection port was 280°C, and the injection volume was 0.2 *μ*L

The condition of mass spectrometry

1. the solvent delay was 8 minutes
2. the temperature of the ion source was 250°C, and the temperature of ports was 280°C
3. MS scanning range was from 40 to 500 Da by M/Z
4. the ionization mode was EI
5. the electron energy was 70 eV

Each sample was injected three times continuously. Every tongue-coating sample processed by the silane derivative method was divided into three identical aliquots for injection to avoid error.

### 2.5. Data Processing, Statistical Analysis, and Substance Identification

Capric acid was used as an internal control for normalization of MS data. Tongue-coating samples without detected internal control were removed. MS data preprocessing including filtering, peak detection, deconvolution, peak alignment, and normalization was performed using the internal software of Thermo Fisher Scientific.

PLS Discriminant Analysis (PLS-DA) in the SIMCA-P+ program was applied to find components to achieve maximum separation between two groups and to understand which variables contribute to group separation. The first and second principle components of samples were chosen for plotting.

The two-sample Welch *t*-statistics and variable importance in the projection (VIP) value analysis [17] were applied to compare the peak value of two groups. Peaks with *P* < 0.05 and VIP ≥ 1 were selected as differential metabolites. Common metabolites were identified from the peaks with *P* > 0.05 and VIP < 1. The metabolites were mined out by searching the METLIN and NIST data according to their mass charge ratio and retention time.

## 3. Results

**Figure 1:**
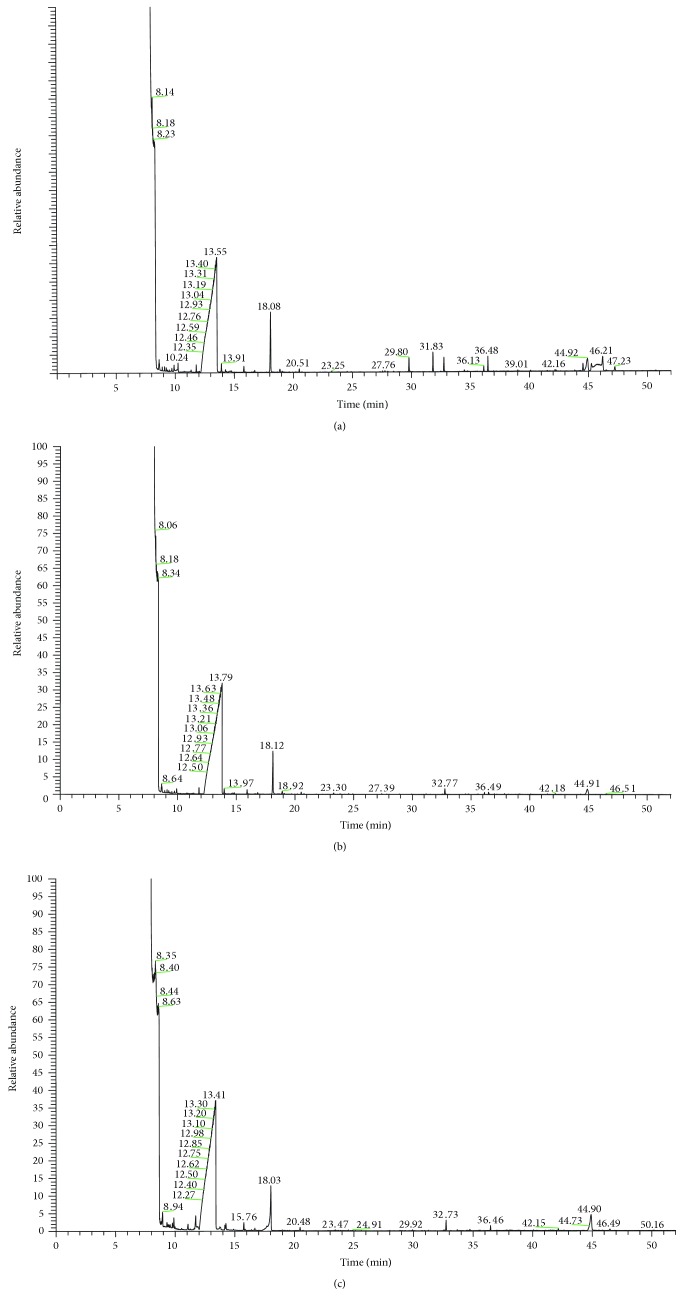
Tongue-coating samples from the healthy subjects and patients with CHD showing the damp phlegm pattern and the nondamp phlegm pattern were examined by GC-MS: (a) CHD damp phlegm pattern, (b) CHD nondamp phlegm pattern, and (c) healthy subjects.

**Figure 2:**
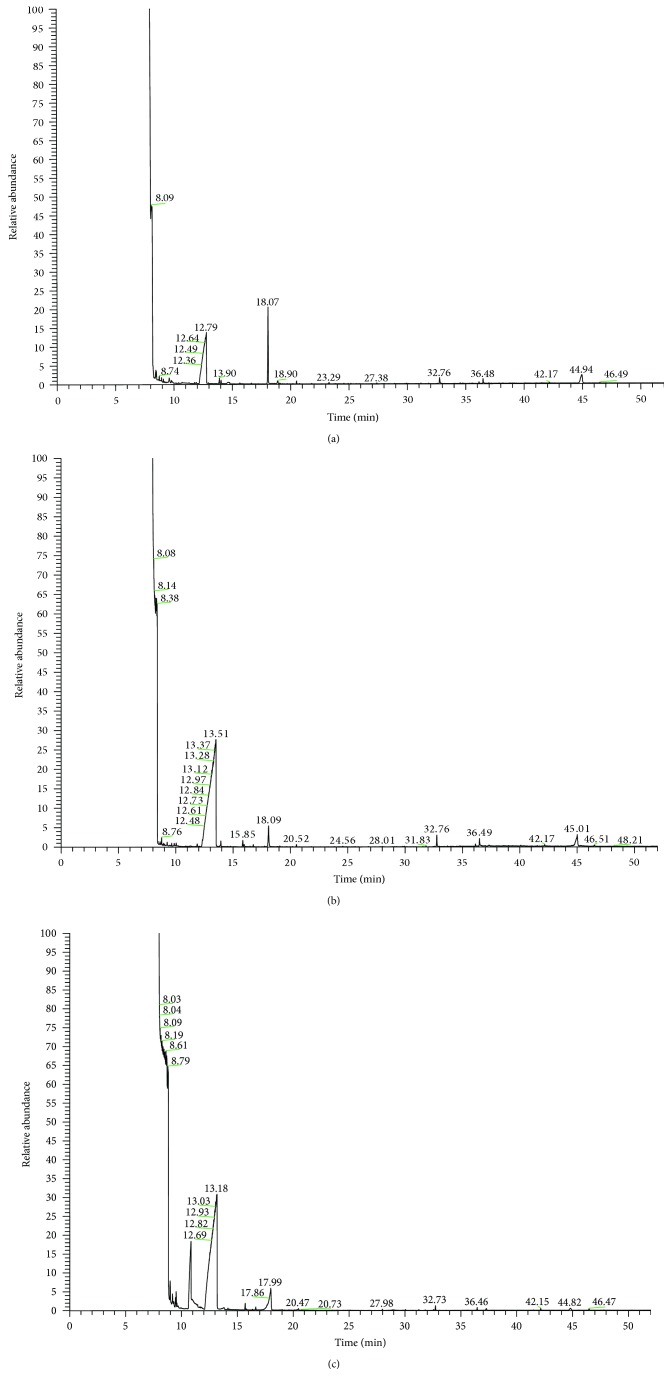
Tongue-coating samples from the healthy subjects and patients with CRF showing the damp phlegm pattern and nondamp phlegm pattern were examined by GC-MS: (a) CRF damp phlegm pattern, (b) CRF nondamp phlegm pattern, and (c) healthy subjects.

Tongue-coating samples from the healthy subjects and samples from patients with CHD/CRF showing the damp phlegm pattern and the nondamp phlegm pattern were examined by GC-MS. Examples of the GC-MS spectra are shown in Figures 1 and 2. As shown in the two figures, there were some different mass spectrum peaks among the healthy subjects, with CHD/CRF showing the damp phlegm pattern and nondamp phlegm pattern patients.

### 3.1. The Discrimination Analysis of Partial Least Squares (PLS-DA)

**Figure 3:**
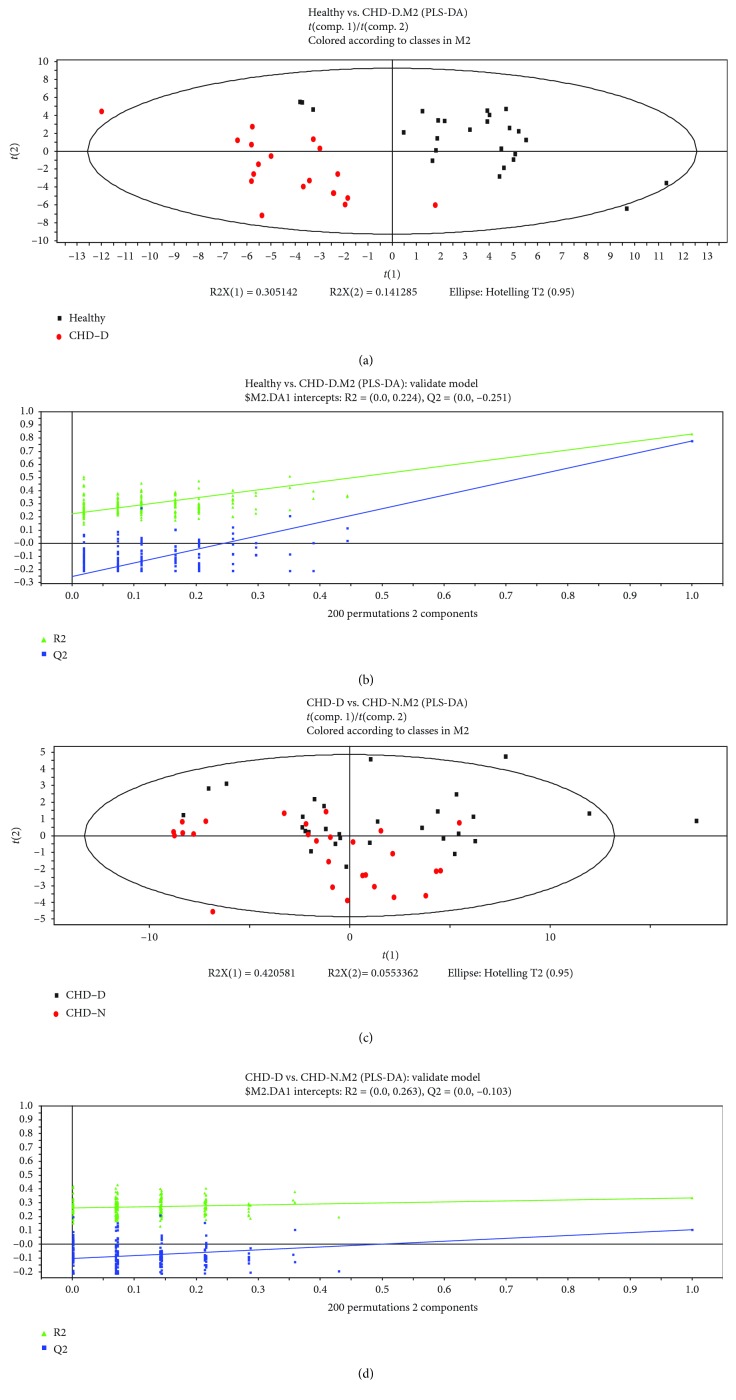
PLS-DA analysis of metabolomic data and validation plots of PLS-DA scores: (a) healthy subjects (black points) vs. CHD damp phlegm pattern (red points), (b) validation plots of PLS-DA scores (healthy subjects vs. CHD damp phlegm pattern), (c) CHD damp phlegm pattern (black points) vs. CHD nondamp phlegm pattern (red points), and (d) validation plots of PLS-DA scores (CHD damp phlegm pattern vs. CHD nondamp phlegm pattern).

**Figure 4:**
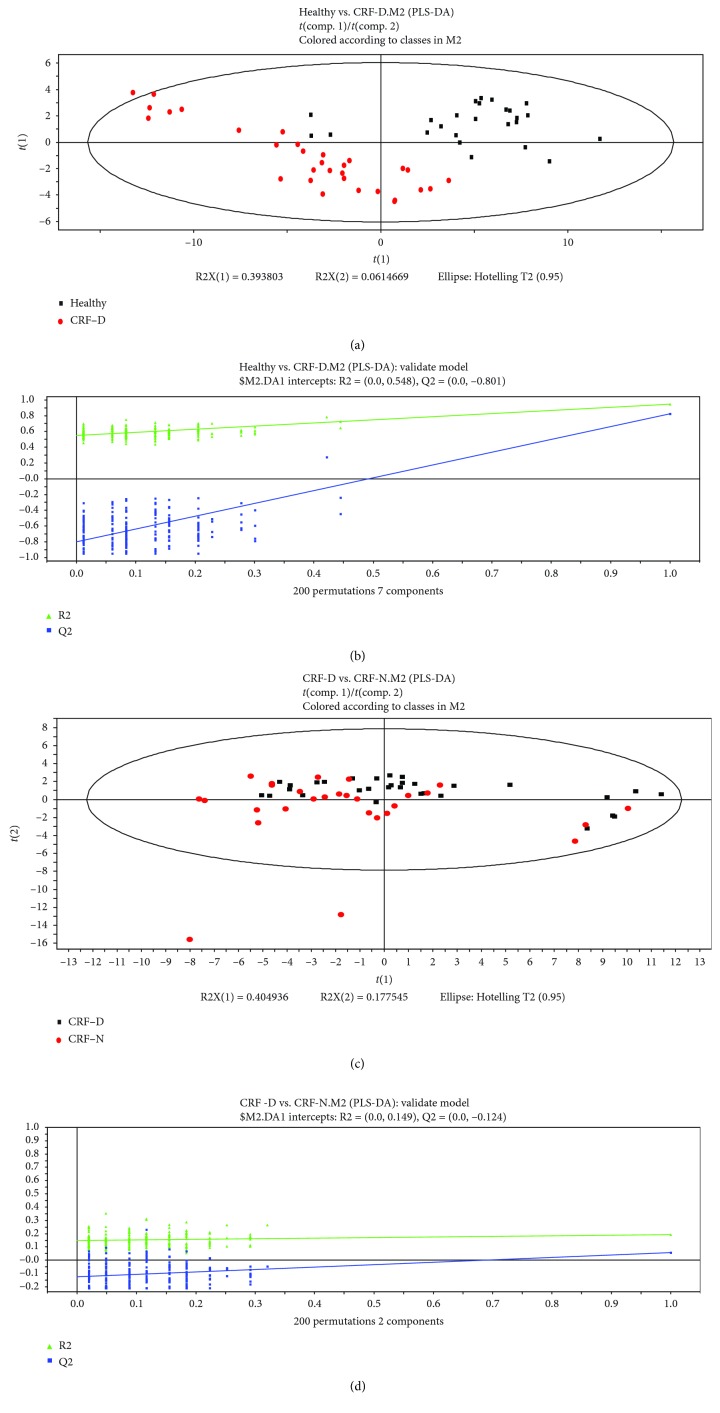
PLS-DA analysis of metabolomic data and validation plots of PLS-DA scores: (a) healthy subjects (black points) vs. CRF damp phlegm pattern (red points), (b) validation plots of PLS-DA scores (healthy subjects vs. CRF damp phlegm pattern), (c) CRF damp phlegm pattern (black points) vs. CRF nondamp phlegm pattern (red points), and (d) validation plots of PLS-DA scores (CRF damp phlegm pattern vs. CRF nondamp phlegm pattern).

As shown in Figures 3(a), 3(b), 4(a) and 4(b), the patients with CHD/CRF can be distinguished clearly from healthy subjects by using the PLS-DA method. The separation of patients with the damp phlegm pattern from the nondamp phlegm pattern was noticeable, although there was still some overlap between these two groups (Figures 3(c), 3(d), 4(c) and 4(d)). It showed that the differences in tongue-coating components between patients with CHD/CRF and healthy subjects were higher than the differences between the damp phlegm pattern group and the nondamp phlegm pattern group.

**Figure 5:**
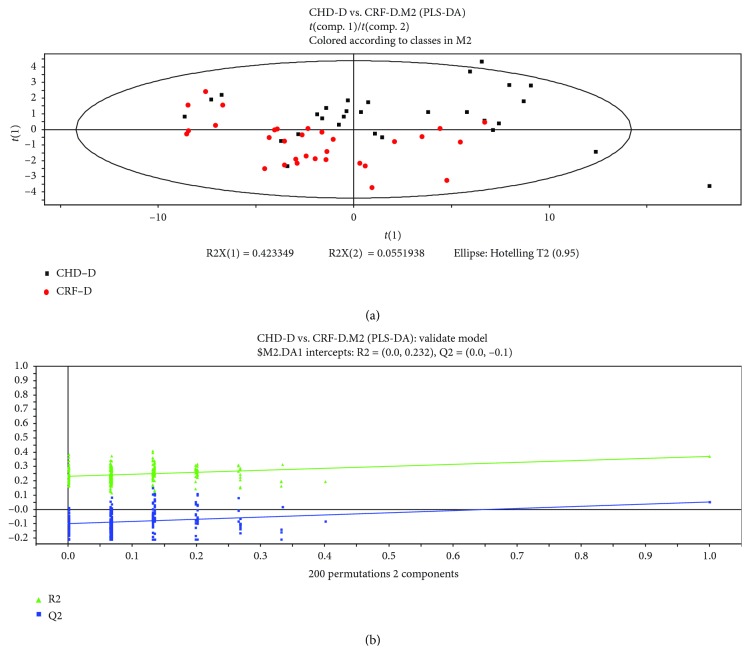
PLS-DA analysis of metabolomic data and validation plots of PLS-DA scores: (a) CHD damp phlegm pattern (black points) vs. CRF damp phlegm pattern (red points); (b) validation plots of PLS-DA scores (CHD damp phlegm pattern vs. CRF damp phlegm pattern).

In addition, Figures 5(a) and 5(b) showed that both the CHD patients and the CRF patients with the damp phlegm pattern also had some overlap, which indicated that there might be some similarities among damp phlegm pattern patients with different diseases.

### 3.2. GC-MS Peaks and Their Corresponding Metabolites

As listed in Table 2, there were nine different peaks existing in the tongue coating of patients with CHD showing the damp phlegm pattern compared to healthy subjects. The corresponding metabolites based on the different peaks were identified through searching the METLIN and HMDB databases. These metabolites included decanoyl-L-carnitine, desoxycorticosterone acetate, L-histidine, androstenedione, pregnanolone, oxoadipic acid, creatinine, D-lactose, and L-tryptophan. Decanoyl-L-carnitine reflects the abnormity of fatty acid oxidation. Desoxycorticosterone acetate is one of the steroid hormones secreted by the fascicular zone of the adrenal cortex, which is involved in the source and storage of energy. These metabolites indicate that there are abnormities of lipid and energy metabolism in CHD patients. Histidine and tryptophan are essential amino acids for humans. Oxoadipic acid is the degradation product of lysine. Lactose is the important composite in the pathway of galactose metabolism, and its appearance could indicate the abnormity of the glucose metabolism for the patients of CHD. The endogenous sex hormones in patients with CHD were also different compared to healthy subjects. It was reported that the ratio of estradiol to testosterone in the serum of male CHD patients increased [18]; meanwhile, the level of estradiol decreased and that of testosterone increased in postmenopausal female CHD patients [19]. Creatinine is also a risk indicator for the patients with the damp phlegm pattern of CHD, which could indicate the aggravation of diseases or the existence of complications.

Similarly, for CRF, nine peaks were identified between patients showing the damp phlegm pattern and healthy subjects. The corresponding metabolites were creatinine, D-lactose, gluconolactone, androstenedione, acetylcarnitine, oxoadipic acid, acetaminophen glucuronide, 7,8-dihydro-L-biopterin, and 3-methoxytyramine. The abnormity of creatinine is a typical manifestation for CRF, which can not only be detected from the blood but also be discovered from the tongue coating of patients. The patients with CRF often have the disorder of glucose metabolism. Their sugar tolerance curve is like that of patients with diabetes. Meanwhile, the patients diagnosed with CRF do not have sufficient gluconeogenesis and may have hypoglycemia during the period of uremia. Therefore, the metabolisms of lactose, gluconolactone, and acetaminophen glucuronide are abnormal. Acetylcarnitine has effects on the neuroprotection, neuromodulation, and neurotrophy. Besides those, it also plays an important role in defending the diseases. Meanwhile, acetylcholine is the source of energy storage and is involved in the metabolism of alanine and aspartic acid. 7,8-Dihydro-L-biopterin is the oxidative product of tetrahydrobiopterin, and it is closely related to the synthesis of catecholamine. It has been shown in many studies that the norepinephrine levels of hemodialysis patients with CRF were significantly higher than those of patients without the disease, which may be related to kidney damage and the metabolism of catecholamine [20]. 3-Methoxytyramine is one of the neurotransmitters. It is an excitatory neurotransmitter and can enhance the renal blood flow. Its metabolism is abnormal in patients with CRF.

Only three (including pyrocatechol, O-phosphorylethanolamine, and L-tyrosine) and four (including 3-hydroxy-DL-kynurenine, uridine, L-proline, and anthranilic acid) peaks were found between damp phlegm pattern and nondamp phlegm pattern patients with CHD and CRF, respectively. The results confirmed that the differences between patients and healthy subjects are higher than the differences between damp phlegm pattern and nondamp phlegm pattern patients.

As a kind of phosphoric monoester, phosphorylethanolamine is a sphingolipid metabolite which is closely related to atherosclerosis. It may be involved in mediating the interaction between low-density lipoprotein and vascular smooth muscle cells, thus activating platelets to promote blood coagulation [21]. Tyrosine is the human body essential amino acids and ketone glucogenic amino acids. Pyrocatechol is part of the metabolism of tyrosine.

3-Hydroxy-DL-kynurenine has neurotoxicity and may cause bladder cancer. Uridine is a component of the nucleic acid of human cells. L-Proline is one of the most important amino acids in the synthesis of human protein. Anthranilic acid is the oxidation product of tryptophan metabolism.

In addition, as shown in Table 3, there were five different (including L-tryptophan, acetylcarnitine, 5-hydroxyindoleacetic acid, acetaminophen glucuronide, and 3-hydroxy-DL-kynurenine) and five common peaks (including *γ*-butyrolactone, imidazoleacetic acid, D-glucuronic acid, L-threonine, and L-aspartic acid) existing in the tongue-coating samples from CHD damp phlegm pattern patients compared to CRF damp phlegm pattern patients. These substances may provide information about the molecular basis of the “different diseases have the same pattern” theory in TCM.

3-Hydroxy-DL-kynurenine is one of the intermediate products in the catabolism of tryptophan. 5-Hydroxyindoleacetic acid is the decomposition product of serotonin, which is a neurotransmitter. Some studies show that the 5-hydroxyindoleacetic acid level is the highest in patients with acute myocardial infarction. Its levels in patients with unstable and stable angina pectoris were relatively lower [22]. All these different metabolites were mainly related to the abnormities of amino acid metabolism, glucose metabolism, and neurotransmitter levels.

Threonine is one kind of essential amino acids for humans, and the levels of threonine were similar in patients with the damp phlegm pattern of CHD and CRF. Although aspartic acid is not an essential amino acid, it is the raw material to synthesize the methionine, threonine, isoleucine, and lysine. It is also involved in the urea cycle to eliminate the toxic amines. D-Glucuronic acid is related to the metabolisms of starch and sucrose.

## 4. Discussion

In this study, we applied the GC-MS technique to identify the tongue-coating metabolites of patients of CHD and CRF. The results showed that, compared with nondamp phlegm pattern patients and healthy subjects, patients with the damp phlegm pattern of CHD had some kind of metabolism abnormity of amino acids, lipids, and glucose, while patients with the damp phlegm pattern of CRF mainly had metabolism abnormities of amino acids and glucose. Three metabolites (pregnanolone, oxoadipic acid, and O-phosphorylethanolamine) were also identified in our previous studies on the serum metabolites of CHD damp phlegm pattern patients compared with nondamp phlegm pattern patients and healthy subjects [23]. The metabolites are related to lysine, endogenous sex hormones, and sphingolipid metabolism. In addition, two proteins (neutral alpha-glucosidase AB and sorting nexin-10) were identified in the tongue coating of CHD damp phlegm pattern patients compared with nondamp phlegm pattern patients and healthy subjects by using the proteomic technique [8]. The neutral alpha-glucosidase AB (gene GANAB) cleaves sequentially the two innermost alpha-1,3-linked glucose residues from the Glc_2_Man_9_GlcNAc_2_ oligosaccharide precursor of immature glycoproteins [24]. This protein is involved in the pathway N-glycan metabolism, which is part of glucose metabolism. And the sorting nexin-10 (gene SNX10) can bind the phosphoinositide, both of which involve lipid metabolism [25]. We also found one protein, kish-A (gene TMEM167A) [6], that was involved in the early part of the protein secretory pathway [26] and was identified in the tongue coating of CRF damp phlegm pattern patients compared with nondamp phlegm pattern patients. Therefore, the results of our previous research on tongue-coating proteomics conformed to the findings of this paper.

In this study, we found five common peaks in the tongue-coating samples from CHD damp phlegm pattern patients compared to CRF damp phlegm pattern patients. According to the etiology and pathogenesis theory of TCM, both CHD and CRF are caused by dampness and they may share common symptoms including heavy sensation in the limbs and body and greasy tongue coating. Our metabolomic results indicate that there were common metabolic abnormities of amino acids and glucose in patients with the damp phlegm pattern of CHD and CRF, which may be the molecular basis of the TCM damp phlegm pattern.

On the other hand, the lesion site of CHD is different from that of CRF. These two diseases also show different manifestations. For example, CHD patients have chest suffocation, chest pain, etc. CRF patients have nausea, vomiting, etc. In this study, five different metabolites were also found in the tongue coatings from the damp phlegm pattern of CHD and CRF, which indicated that the damp phlegm pattern in CHD and CRF might have different pathogeneses.

In the future, more patient samples will be collected to validate the identified metabolites using different approaches. We will explore the possibility of using these metabolites as novel biomarkers for early detection of disease.

## 5. Conclusion

In our study, some important metabolites were identified in tongue-coating samples of the TCM damp phlegm pattern of CHD and CRF by metabolomic technique. Metabolites identified in this study may be used as noninvasive and convenient biomarkers for distinguishing the TCM damp phlegm pattern of CHD and CRF patients. Our results also provide important information to understand the TCM theory of “different diseases have the same pattern.”

## Figures and Tables

**Table 1:** Summary of demographics and clinic characteristics of CHD patients, CRF patients, and healthy subjects.

Group	Cases	Ratio of male to female	Average age	Average course of disease (year)
Healthy subjects	25	1 : 0.79	53.92 ± 4.66	N/A
CHD showing the damp phlegm pattern	29	1 : 0.81	72.3 ± 12.67	5.90 ± 2.48
CHD showing the nondamp phlegm pattern	29	1 : 0.61	69.41 ± 12.45	5.66 ± 2.81
CRF showing the damp phlegm pattern	32	1 : 0.60	53.37 ± 17.18	3.66 ± 1.62
CRF showing the nondamp phlegm pattern	30	1 : 1.14	52.16 ± 14.21	3.67 ± 1.79

**Table 2:** Different peaks and metabolites identified from the tongue-coating samples.

*M*/*Z*	RT	VIP	*P*	Formula	Metabolites
*CHD showing the damp phlegm pattern compared to healthy subjects*					
315.2410	2167.8	1.60	9.73*E* − 08	C_17_O_33_NO_4_	Decanoyl-L-carnitine
372.2301	2528.4	1.57	6.21*E* − 06	C_23_H_32_O_4_	Desoxycorticosterone acetate
155.0695	529.2	1.56	6.73*E* − 09	C_6_H_9_N_3_O_2_	L-Histidine
286.1933	1948.2	1.56	7.09*E* − 08	C_19_H_26_O_2_	Androstenedione
318.2559	590.4	1.54	5.96*E* − 03	C_21_H_34_O_2_	Pregnanolone
160.0372	2172.0	1.53	2.79*E* − 06	C_6_H_8_O_5_	Oxoadipic acid
113.0589	826.2	1.40	9.00*E* − 05	C_4_H_7_N_3_O	Creatinine
342.1126	2340.0	1.35	6.73*E* − 06	C_12_H_22_O_11_	D-Lactose
204.0899	1909.2	1.22	3.20*E* − 03	C_11_H_12_N_2_O_2_	L-Tryptophan
*CHD showing the damp phlegm pattern compared to the nondamp phlegm pattern*					
110.0368	568.2	2.49	4.18*E* − 02	C_6_H_6_O_2_	Pyrocatechol
141.0191	940.2	2.32	4.24*E* − 02	C_2_H_8_NO_4_P	O-Phosphorylethanolamine
181.0739	948.0	2.31	4.96*E* − 02	C_9_H_11_NO_3_	L-Tyrosine
*CRF showing the damp phlegm pattern compared to healthy subjects*					
113.0589	824.4	1.69	2.00*E* − 05	C_4_H_7_N_3_O	Creatinine
342.1126	2340.0	1.57	2.20*E* − 06	C_12_H_22_O_11_	D-Lactose
178.0477	705.6	1.43	3.00*E* − 05	C_6_H_10_O_6_	Gluconolactone
286.1933	1947.6	1.43	5.43*E* − 08	C_19_H_26_O_2_	Androstenedione
203.1158	1800.0	1.27	5.35*E* − 03	C_9_H_17_NO_4_	Acetylcarnitine
160.0372	2172.0	1.26	2.16*E* − 07	C_6_H_8_O_5_	Oxoadipic acid
327.0954	1131.6	1.25	2.00*E* − 07	C_14_H_17_NO_8_	Acetaminophen glucuronide
239.1018	694.2	1.23	1.89*E* − 03	C_9_H_13_N_5_O_3_	7,8-Dihydro-L-Biopterin
167.0946	568.2	1.21	1.08*E* − 06	C_9_H_13_NO_2_	3-Methoxytyramine
*CRF showing the damp phlegm pattern compared to the nondamp phlegm pattern*					
224.0797	592.8	1.81	1.67*E* − 03	C_10_H_12_N_2_O_4_	3-Hydroxy-DL-kynurenine
244.0695	516.6	1.35	2.13*E* − 02	C_9_H_12_N_2_O_6_	Uridine
115.0633	580.2	1.22	1.93*E* − 02	C_5_H_9_NO_2_	L-Proline
137.0477	528.6	1.19	4.20*E* − 02	C_7_H_7_NO_3_	Anthranilic acid

**Table 3:** Different/common peaks and metabolites identified from the tongue-coating samples.

*M*/*Z*	RT	VIP	*P*	Formula	Metabolites
*Different peaks and metabolites of CHD showing the damp phlegm pattern compared to CRF showing the damp phlegm pattern*					
204.0899	1909.8	2.57	4.76*E* − 02	C_11_H_12_N_2_O_2_	L-Tryptophan
203.1158	1803.0	2.65	4.10*E* − 02	C_9_H_17_NO_4_	Acetylcarnitine
191.0582	1920.0	2.35	1.21*E* − 03	C_10_H_9_NO_3_	5-Hydroxyindoleacetic acid
327.0954	1140.0	1.81	2.21*E* − 02	C_14_H_17_NO_8_	Acetaminophen glucuronide
224.0797	592.8	1.36	1.09*E* − 02	C_10_H_12_N_2_O_4_	3-Hydroxy-DL-kynurenine
*Common peaks and metabolites of CHD showing the damp phlegm pattern compared to CRF showing the damp phlegm pattern*					
86.0368	600.0	0.13	1.49*E* − 01	C_4_H_6_O_2_	*γ*-Butyrolactone
126.0429	853.8	0.24	2.66*E* − 01	C_5_H_6_N_2_O_2_	Imidazoleacetic acid
194.0427	1033.8	0.34	2.05*E* − 01	C_6_H_10_O_7_	D-Glucuronic acid
119.0582	2701.8	0.34	6.06*E* − 01	C_4_H_9_NO_3_	L-Threonine
133.0375	2718.0	0.42	5.60*E* − 01	C_4_H_7_NO_4_	L-Aspartic acid

## Data Availability

The measurement data used to support the findings of this study are restricted by the Ethics Committee of the Shanghai University of Traditional Chinese Medicine in order to protect patient privacy. Data are available from Yiming Hao (Email: hymjj888@163.com) for researchers who meet the criteria for access to confidential data.
